# Frailty transitions in electronic health records: who first? what first?

**DOI:** 10.18632/aging.206247

**Published:** 2025-05-12

**Authors:** Fabienne Hershkowitz Sikron, Rony Schenker, Orit Shahar, Achinoam Ben Akiva-Maliniak, Galit Segal, Yishay Koom, Idit Wolf, Bawkat Mazengya, Maor Lewis, Tzippy Shochat, Dov Albukrek

**Affiliations:** 1 Department of Epidemiology and Medical Quality Assessment, Meuhedet HMO, Tel-Aviv Yaffo 6203854, Israel; 2 Director of Knowledge Development and Research, Joint-Eshel, 9 Eliezer Kaplan, Jerusalem 9103401, Israel; 3 Director, Rehabilitation and Preservation of Functionality, Joint-Eshel, 9 Eliezer Kaplan, Jerusalem 9103401, Israel; 4 Coordinator of Preservation of Functionality, Joint-Eshel, 9 Eliezer Kaplan, Jerusalem 9103401, Israel; 5 Chief Geriatric Physician Meuhedet HMO, Tel-Aviv Yaffo 6203854, Israel; 6 Director, Senior Citizen Department, Meuhedet HMO, Tel-Aviv Yaffo 6203854, Israel; 7 Chief Geriatric Nurse, Meuhedet HMO, Tel-Aviv Yaffo 6203854, Israel; 8 Data Analytics, Meuhedet HMO, Tel-Aviv Yaffo 6203854, Israel; 9 MD, Medical Division, Meuhedet HMO, Tel-Aviv Yaffo 6203854, Israel; 10 Research Institute, Meuhedet HMO, Tel-Aviv Yaffo 6203854, Israel; 11 Chief Medical Officer, Meuhedet HMO, Tel-Aviv Yaffo 6203854, Israel

**Keywords:** frailty transition, electronic frailty index, older people, health maintenance organization, statistics and numerical data

## Abstract

Background: Frailty is associated with an increased risk of adverse health outcomes and may worsen over time.

Objectives: This study aims to describe the dynamic trajectory of frailty, identify the characteristics of those who deteriorate first, and determine what deteriorates first.

Study Design and Setting: A primary care longitudinal population-based cohort with repeated measures at baseline and one year later.

Participants: The cohort included all 119,952 Meuhedet members aged 65 years and over as of January 2023.

Predictors: Demographic factors, health indicators, and the Meuhedet Electronic Frailty Index containing 36 deficits.

Outcomes: Worsening frailty is defined as a higher frailty level one year later in 2024 compared to 2023. A new frailty deficit is defined as a deficit appearing in 2024 that was not present in 2023.

Statistical Analysis: The comparison of worsening percentages by demographic and clinical characteristics was tested using the chi-square test at the univariable level and logistic regression at the multivariable level.

Results: Overall, 13.3% of participants worsened after one year of follow-up, with 2.3% dying. Higher risk groups for worsening included females, older individuals, those belonging to the Arab sector, and those with multimorbidity. New deficits mainly included modifiable risk factors related to general health and functionality, despite chronic diseases being more frequent at baseline.

Conclusions: Emphasizing intervention programs based on these health promotion issues may significantly impact disease control and slow frailty worsening.

## INTRODUCTION

Emerging evidence suggests significant variability in the health status of older individuals, with people of the same age differing greatly in their vulnerability to adverse outcomes [1]. This variability is often referred to as frailty [2]. Geriatricians define frailty as a biological syndrome of decreased reserve and resistance to stressors, resulting from cumulative declines across multiple physiological systems, causing vulnerability to adverse outcomes [3]. Frailty is associated with an increased risk of adverse health consequences, including falls [4], hospitalization [5, 6], and death [2, 7–9].

Based on the cumulative deficit model of Rockwood and Mitnitski [10, 11], an Electronic Frailty Index (EFI) has been developed and validated, allowing the classification of patients according to their level of frailty [12]. Today, the EFI is routinely adopted within all UK primary care settings [13, 14]. Since the operative definitions of such an index are specific to each country [15, 16], versions of the EFI have been developed in various countries, including the US [8, 17], Canada [18], Australia [19], China [20], Japan [21], Sweden [22, 23], and other parts of the United Kingdom such as Wales [24] and Scotland [25].

Frailty is not static; it is a dynamic health state that changes over time, even within a relatively short 12 months follow-up period [26]. It usually worsens but may also improve [2]. Despite the importance of the dynamic nature of frailty and its association with increased disability in terms of ADL [27], increased use of health care services [28], and all-cause mortality [29], studies on predictors of frailty worsening over time are sparse in the general geriatric literature [30, 31]. A meta-analysis by Kojima et al. showed pooled rates of frailty transition patterns among community-dwelling older people from 16 cohorts [32]. They found an association between older age and frailty worsening [33–35], and that women were more likely to change frailty status, either improving or worsening, rather than staying the same. Greater frailty at baseline increased the likelihood of worsening at follow-ups [36]. Multimorbidity was associated with frailty worsening among non-frail participants [31, 33], as well as polypharmacy [37, 38] and lower self-rated health [36, 39]. Physical inactivity [40], mobility impairment [39], and slow gait speed [26, 41] were also associated with frailty worsening. Social predictors such as fewer social interactions, living alone [42, 43], low education [31, 35, 44, 40], difficulty meeting living expenses [39], and being part of a minority [45] were also identified as predictors of frailty worsening. Other predictors of frailty worsening included psychological predictors such as depressed mood [46], sensory variables such as visual and hearing impairment [39], decreased cognitive activities [47], and cognitive impairment [37, 39].

Recently, our team developed an EFI according to our needs as an HMO, called MEFI (Meuhedet Electronic Frailty Index). MEFI contains 36 deficits, based on Clegg’s items [12] and Orkaby’s items [8]. MEFI was validated and has been proven to predict hospitalization and mortality [6], and was used to measure frailty and frailty worsening in this study.

It was found that an index based on the cumulative deficit model, such as the MEFI, better captures the multidimensional and dynamic nature of frailty over time [40], is considered a more accurate predictor of mortality [7], and is more sensitive to modifications in underlying health than the phenotype model [48]. A look at the predictors of frailty worsening, such as multimorbidity, activity limitation, or sensory impairment, reveals that they are all represented by one or more specific deficits included in MEFI (see Table 1). Using MEFI deficits to define predictors of frailty worsening is advantageous since they are routinely collected anyway, and the definition of the deficits is quite agreed upon beyond the various EFI in use in the literature. Unfortunately, no studies on predictors of frailty worsening, in terms of EFI deficits, could be found.

**Table 1:** List of 36 deficits included in the MEFI.

**Deficits**
Activity Limitation
Anaemia and Haematinic Deficiency
Anxiety
Arthritis
Atrial Fibrillation
Cancer (any except basal cell skin cancer)
Cerebrovascular Disease
Chronic Kidney Disease
Coronary Artery Disease
Dementias
Depression
Diabetes
Dizziness/Vertigo
Fall/fall-related injuries (hip/skull fractures, subdural hematoma)
Fatigue
Gait Abnormality
Gastro-intestinal Disease
Hearing Impairment
Heart Failure
Housebound
Hypertension
Lung Disease
Memory and Cognitive Problems
Muscular Wasting
Osteoporosis
Parkinson’s Disease
Peripheral Neuropathy
Peripheral Vascular Disease
Polypharmacy
Requires Care
Sleep Disturbance
Social Vulnerability
Thyroid Disease
Urinary Incontinence
Vision Comorbidity
Weight Loss in the past year

The objectives of this study are (1) to describe the dynamic trajectory of frailty, (2) to identify the characteristics of those who deteriorate first, and (3) to identify which deficits deteriorate first in each frailty level. A better understanding of frailty worsening among community-dwelling older adults will help define early warning indicators of who will worsen first and determine preventive measures focused on what will worsen first.

## RESULTS

### Participants

The cohort included all 119,952 patients of the Meuhedet HMO aged 65 and over, 54.4% of whom were females (See Table 2). The largest age group was those aged 65–74, with a mean age of 73.8 (SD = 7.0), a median of 72, a range from 65 to 106, and an interquartile range of 68 to 78. More than half belonged to the middle social level, and 8.3% belonged to the Arabic sector. Regarding frailty levels, 37.4% were fit, 40.3% were mildly frail, 16.8% were moderately frail, and 5.5% were severely frail. Regarding other aspects of their medical condition, 16.7% were hospitalized at least once the year before follow-up, 7.4% had a CCI score higher than 5, and 70% were overweight or obese.

**Table 2:** Baseline characteristics, worsening rates and crude OR.

***N* = 119,952**	**Distribution at BL**	**Pct. worsened**
**All**	**100% *N* = 119,952**	within each sub-group^**^
**Sex**		
Male	45.6%	13.1%^*^
Female	54.4%	13.5%
**Age groups**		
65–74 years	60.8%	10.0%
75–84 years	29.8%	16.2%
85+ years	9.3%	25.6%
SES groups		
**Low**	25.3%	14.5%
Intermediate	54.1%	13.3%
High	20.6%	11.6%
**Sector**		
Jewish secular	77.4%	13.2%
Jewish orthodox	14.2%	12.5%
Arabic	8.3%	15.9%
**MEFI 2023**		
Fit	37.4%	11.9%
Mild frailty	40.3%	13.3%
Moderate frailty	16.8%	18.0%
Severe frailty	5.5%	8.9%
**Hosp. year before**		
No	83.3%	12.3%
Yes	16.7%	18.6%
**CCI groups**		
0	31.4%	8.4%
1–2	35.1%	13.5%
3–5	26.1%	16.8%
6+	7.4%	21.1%
**BMI groups**		
Underweight	1.6%	21.2%
Normal weight	28.4%	13.2%
Overweight	39.4%	12.6%
Obesity	30.7%	14.0%
**Top 15 deficits**		
Polypharmacy	89.2%	14.3%
Hypertension	72.9%	15.0%
Arthritis	55.3%	15.2%
Diabetes	32.7%	15.7%
Social vulnerability	27.5%	16.6%
Lung disease	24.8%	15.8%
Memory/cognitive	24.1%	19.6%
PVD	23.7%	17.0%
Coronary Artery	22.2%	16.8%
GI disease	22.2%	14.9%
Thyroid disease	21.1%	14.7%
Cancer	21.1%	16.9%
Cerebrovascular TI	18.3%	17.5%
Kidney	14.1%	19.1%
Atrial Fibrillation	12.1%	19.7%

^*^Pearson Chi-Square, *p* < 0.05. ^**^Pearson Chi-Square, *p* < 0.001.

### Worsening and frailty transitions

**Figure 1:**
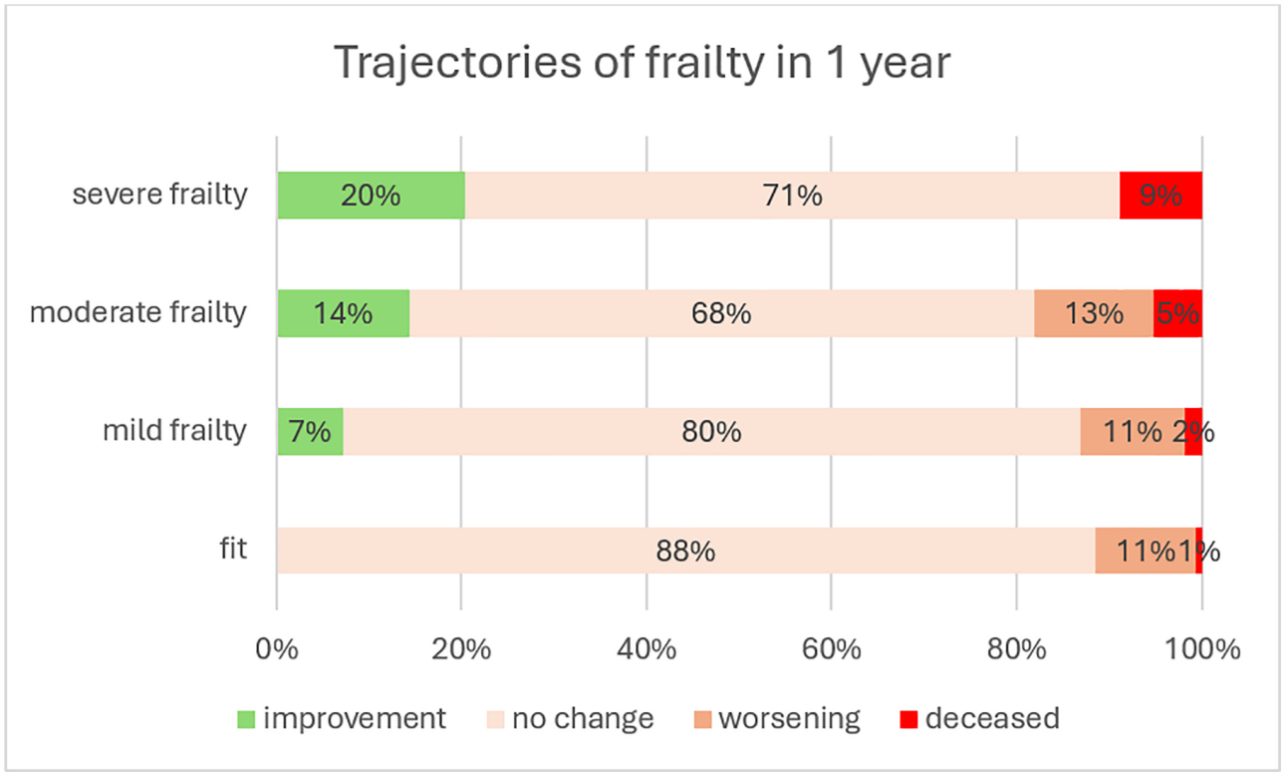
Trajectories of frailty in 1 year.

Overall, 13.3% of the cohort experienced worsening of their MEFI after one year of follow-up, and 2.3% had died. The worsening rate, including those who were deceased, was 11.9%, 13.3%, 18.0%, and 8.9% among the fit, mildly frail, moderately frail, and severely frail, respectively. The deceased rate was 0.6%, 1.9%, 5.2%, and 8.9%, respectively (see Figure 1). Estimated transitions from fit to any level of frailty were 10.2% for those aged 65–74, 17.2% for those aged 75–84, and 32.2% for those aged 85+. The worse the frailty was at baseline, the higher the percentage of dying. In each frailty level at baseline, people were likely to remain in their current frailty category, and transitions between adjacent frailty levels were more frequent than those across several frailty levels.

### Predictors of worsening - WHO will worsen first

The worsening rate, including those who died, was 13.5% for females and 13.1% for males (*p* < 0.001). It increased with age, from 10.0% to 16.2% and 25.6% among the age groups 65–74, 75–84, and 85+, respectively (*p* < 0.001). The worsening rate decreased as socio-economic status increased, from 14.5% to 11.6%, and was higher among the Arabic sector (15.9%) compared to the general secular Jewish sector (13.2%) (*p* < 0.001). The worsening rate increased with frailty level at baseline, from 11.9% among the fit to 13.3% among the mildly frail, 18.0% among the moderately frail, and then declined to 8.8% among the severely frail (*p* < 0.001). The death rate increased with frailty level, at 0.6%, 1.9%, 5.2%, and 8.8% among the fit, mild, moderate, and severe frailty groups, respectively (worsening among the severe frailty group means they died). Those who had a previous hospitalization during the year 2022 had a worsening rate of 18.6%, a 52% higher rate than those who were not hospitalized (*p* < 0.001). Higher CCI scores showed a higher worsening rate, from 8.4% to 13.5%, 16.8%, and 21.1% for the fit, mild frailty, moderate frailty, and severe frailty, respectively (*p* < 0.001). The baseline deficits in January 2023 most associated with worsening, with a crude OR >1.5 or lower than 0.5, were, in decreasing order: polypharmacy, dementia, housebound, heart failure, memory and cognitive problems, hypertension, atrial fibrillation, kidney diseases, fall-related, activity limitation, sleep disturbance, and requirement for care.

A multivariate analysis conducted to determine factors associated with frailty worsening revealed that being female (OR = 1.18; 95% CI: 1.13, 1.22), being older (aged 75–84 vs. age 65–74: OR = 1.72; 95% CI: 1.65, 1.80); aged 85+ vs. age 65–74: OR = 2.80; 95% CI: 2.63, 2.98), and belonging to the Arabic sector (OR = 1.11; 95% CI: 1.04, 1.19), were associated with increased odds of frailty worsening at 1 year (see Table 3). Conversely, socio-economic status was associated with decreased odds of frailty worsening (SES intermediate vs. low: OR = 0.91; 95% CI: 0.87, 0.95); (SES high vs. low: OR = 0.77; 95% CI: 0.72, 0.82). Higher frailty was associated with lower odds of worsening, compared with the fit level (mild: OR = 0.33; 95% CI: 0.32, 0.35); (moderate 65–74: OR = 0.15; 95% CI: 0.14, 0.16); (severe: OR = 0.02; 95% CI: 0.02, 0.02). The underweight group (OR = 0.56; 95% CI: 0.50, 0.64), the overweight group (OR = 0.53; 95%CI: 0.47, 0.60), and the obese group (OR = 0.58; 95% CI: 0.51, 0.66) had higher odds of worsening, compared with normal weight. Hospitalization during the year before, and higher CCI score, were also predictors for a worsened frailty transition after 1 year (OR = 1.41; 95% CI: 1.35, 1.48). The deficits in 2023 with a crude OR higher than 1.5 or lower than 0.5 at the univariable level were also included in the model. Risk groups with the highest odds of worsening, in descending order, were those with polypharmacy, memory and cognitive problems, housebound, dementia, hypertension, heart failure, and atrial fibrillation. The c-index for discrimination was 0.734, as measured by Harrell’s concordance index (CI95%: 0.729–0.738). There was no multicollinearity between the predictors, with variance in inflation factors (VIFs) less than 3.

**Table 3:** Multivariate logistic regression models for frailty worsening 1 year later.

	**Crude OR**	aOR^*^	95% CI-OR^**^	**Sig**
**Sex**				
Male	Reference	Reference		
Female	1.03	1.18	1.13–1.20	<.001
**Age groups**				
65–74 years	Reference	Reference		
75–84 years	1.62	1.72	1.65–1.79	<.001
85+ years	2.56	2.80	2.63–2.98	<.001
**SES groups**				
Low	Reference	Reference		
Intermediate	0.91	0.91	0.87–0.95	<.001
High	0.80	0.77	0.72–0.82	<.001
**Sector**				
Jewish secular	Reference	Reference		
Jewish orthodox	0.95	0.91	0.86–0.96	<.001
Arabic	1.21	1.11	1.04–1.19	<.001
**MEFI 2023**				
Fit	Reference	Reference		
Mild frailty	1.12	0.57	0.54–0.60	<.001
Moderate frailty	1.51	0.45	0.42–0.48	<.001
Severe frailty	0.74	0.12	0.10–0.13	<.001
**Hosp. year before**				
No	Reference	Reference		
Yes	1.52	1.41	1.35–1.48	<.001
**CCI groups**				
0	Reference	Reference		
1–2	1.62	1.74	1.66–1.83	<.001
3–5	2.01	2.57	2.42–2.73	<.001
6+	2.52	4.01	3.68–4.37	<.001
**BMI groups**				
Underweight	1.61	0.56	0.50–0.64	<.001
Normal weight	Reference	Reference		
Overweight	0.96	0.53	0.47–0.60	0.42
Obesity	1.06	0.58	0.51–0.66	<.001
**Comorbidity**				
Act. Limitation	0.34	0.33	0.25–0.43	<.001
Atrial Fibrillation	1.58	1.59	1.51–1.68	<.001
Dementia	2.03	1.734	1.63–1.86	<.001
Fall Related	0.63	0.61	0.54–0.69	<.001
Heart Failure	1.74	1.60	1.51–1.70	<.001
Housebound	2.00	1.83	1.71–1.97	<.001
Hypertension	1.68	1.66	1.58–1.74	<.001
Kidney disease	1.54	1.39	1.32–1.47	<.001
Memory/Cognitive	1.73	2.23	2.13–2.33	<.001
Polypharmacy	2.75	2.87	2.63–3.13	<.001
Require for Care	0.10	0.05	0.02–0.13	<.001
Sleep disturbance	0.29	0.33	0.27–0.41	<.001

^*^aOR, odds ratio adjusted for other listed variables. ^**^All the aOR were significant at p < 0.001.

### New deficits - WHAT will worsen first

Among the 117,141 patients alive at the end of the follow-up, 38.0% were fit, 40.5% had mild frailty, 16.3% had moderate frailty, and 5.1% had severe frailty at the beginning of the follow-up.

**Figure 2:**
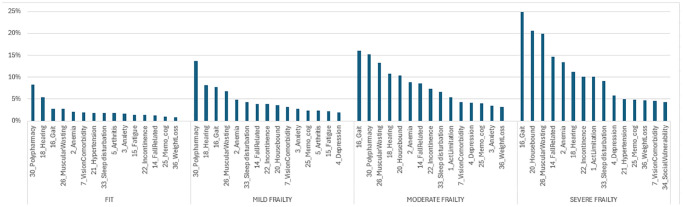
Top 15 new deficits in 2024, stratified by baseline frailty.

At baseline, chronic diseases had the highest prevalence in all frailty groups. Among the fit, mild frailty, moderate frailty, and severe frailty groups, hypertension was reported in 48.9%, 83.2%, 93.7%, and 97.7%, respectively, followed by arthritis in 31.8%, 63.7%, 78.5%, and 88.0%, respectively. Other chronic diseases such as diabetes, lung disease, peripheral vascular disease, and coronary artery disease were also prevalent in each frailty group. However, when looking at the prevalence of new deficits that emerged during the follow-up year, in descending order, most chronic diseases appeared in the lower half of the list. Figure 2 summarizes the top 15 new deficits stratified by frailty. Eight of the deficits appeared in all four frailty groups: gait abnormality, hearing impairment, muscular wasting, anemia, sleep disturbance, incontinence, vision comorbidity, and memory and cognitive problems. The percentage of new deficits increased with frailty level; for example, gait abnormality appeared as a new deficit in 3% of the fit, 8% of the mild, 16% of the moderate, and 25% of the severe frailty groups. Hearing impairment appeared as a new deficit in 6% of the fit, 8% of the mild, and 11% of the moderate and severe frailty groups. When stratifying by age at baseline within each frailty level, most of the top 15 deficiencies beyond age also appeared in each age group. The number of deficits in common with the top 15 list was 14, 14, and 12 among the mild group, 14, 13, and 11 among the mild frailty group, 14, 15, and 14 among the moderate frailty group, and 13, 14, and 15 among the severe frailty group, within those aged 65–74, 75–84, and 85+, respectively (see Supplementary Table 1).

## DISCUSSION

Our paper aims to better understand the dynamics of worsening frailty, focusing on the scope of the issue, identifying who deteriorates first, and determining which deficits are likely to deteriorate first. The ultimate goal is to enhance the quality of care we provide for our patients. By understanding the frequency of frailty deterioration, we can grasp the urgency of taking action. Knowing who deteriorates first allows us to focus our efforts, and identifying what deteriorates first helps us prioritize issues for intervention. Preventing frailty deterioration can be beneficial at the individual clinical care level, in population management by identifying risk groups, and in developing intervention programs that address the most likely problems.

Our first objective was to describe the dynamic trajectory of frailty over one year of follow-up. We found that 12% of fit individuals worsened, and among those with mild frailty, 7% improved, 80% remained in the same frailty category, and 13% worsened. This outcome is very similar to the worsening rate observed in Thompson’s cohort, which was computed to an annual transition rate by Kaskirbayeva [31, 33]. Estimated transitions from fit to any level of frailty were higher in the Walsh cohort, likely due to the younger age of the cohort in our study, but the rates were proportionally almost identical to our cohort [45]. The similarity with these cohorts, which also used community-dwelling participants and an EFI to measure frailty, strengthens and validates our worsening measure. Our second objective was to identify who deteriorates first. We found that 13.3% worsened after one year of follow-up, with higher worsening prevalence among females, older individuals, those with lower socio-economic levels, and those with comorbidities. Our findings align with the literature, including the debate about the association between sex and deterioration. Kojima’s systematic review and meta-analysis found that women change more in both directions [7]. This is consistent with our findings: a higher rate of worsening among females than males (13.5% vs. 13.1%, respectively) and a higher rate of improvement among females than males (7.0% vs. 5.8%, respectively). We also observed that the worsening rate increased with the frailty level, consistent with the literature, but we noted a decline among the severe frailty group. This aligns with the finding that the longest period spent within the same frailty category is among the severely frail [45]. Although a negative relationship between baseline frailty and frailty worsening was found in the multivariate analysis, the positive relationship observed in the univariate analysis reappeared when the model was not controlled for age and comorbidity. Our third objective was to identify what deteriorates first at each frailty level. Among patients alive at the end of follow-up, although chronic diseases had the highest prevalence at baseline, there were few new cases of chronic diseases. This can be explained by the evidence that chronic diseases mostly occur before age 65 and, once diagnosed, remain. A study on the age of onset of chronic diseases showed that the median age of onset for seven diseases (hypertension, diabetes, lung disease, heart disease or stroke, arthritis, neurological diseases, and cancer) was before age 60 [49]. Most new deficits added during follow-up were related to general health and functionality and were similar across the four frailty groups. Even when stratified by age groups, the same new deficits appeared in each age group within the frailty levels. The new MEFI deficits, similar to risk factors found in the literature, included declines in mobility and stability (activity limitation, gait, muscular wasting, and falls), sensory impairment (hearing impairment and vision comorbidity), emotional problems (depression), memory and cognitive impairment, and other signs (sleep disturbance, incontinence). These are modifiable risk factors, and appropriate intervention programs may reduce deterioration. A primary care-based intervention found that a multifaceted approach (physical, nutritional, neurocognitive, and pharmacological) was effective in reversing frailty measures both short-term and at 18 months [50]. A systematic review showed that exercise training can reduce frailty levels and improve prognosis among older adults [51]. A meta-analysis of 15 studies found that resistance band exercise reduced frailty among older adults after 24 weeks [52]. Preventing frailty worsening is crucial due to its association with diseases. Progression from robust to frailty or pre-frailty increased the risk of new-onset diabetes [53] and incident cardiovascular diseases [54]. Moreover, patients who recovered to robust or pre-frail status had decreased risks of incident cardiovascular disease [54]. These findings suggest that reducing frailty has a further impact on reducing adverse outcomes.

The strength of our study lies in its large population-based design with real-world data, exploring how frailty status changes over time, an issue that remains largely unexplored. To our knowledge, this study is the first to specify which deficits may appear first in terms of EFI deficits. In many health systems, these EFI deficits are routinely documented, enabling easy ongoing monitoring. Longitudinal frailty information at the population level is needed to plan services [45]. Specifically, identifying demographic and health risk groups will allow us to determine whom to intervene with first, and identifying deficits at risk for deterioration will help us focus on preventing or delaying frailty transition.

As an HMO, one of our roles is to prevent diseases and improve the health of our patients. Among the population aged 65+, it is essential to understand how modifiable risk factors such as sensory, functional, emotional, and cognitive factors impact frailty worsening, which in turn affects adverse outcomes. Focusing on intervention programs that address these health promotion issues can significantly contribute to disease control and slow the progression of frailty.

### Limitation

One may argue that one year of follow-up is short. However, in high-aged individuals, a one-year observation period seemed sufficient to analyze frailty transition effectively [32]. Another limitation is the inherent limitations of administrative databases and the retrospective nature of this study, which may have led to the incorrect omission of certain deficits. As a result, there may be some random under-reporting, but this would be consistent across the two years compared. A third limitation concerns the length of the look-back period for chronic diseases (from age 55) compared to the commonly accepted one- to three-year period, which may have resulted in an overestimation of certain deficits. However, the decision to use a longer period was driven by coding practices and computational limitations in Meuhedet’s EMR. Since chronic conditions often go uncoded in problem lists, a shorter look-back period could have led to the omission of various chronic conditions.

## CONCLUSIONS

Frailty tends to worsen over time, but the process can be slowed with relevant prevention programs and treatment. Although chronic diseases in old age are frequent, they usually appear earlier in life, and new deficits that may appear later mainly include modifiable risk factors related to general health and functionality. Emphasizing intervention programs based on these health issues may significantly impact disease control and slow frailty worsening.

## MATERIALS AND METHODS

In this study, we adhered to the STROBE reporting guideline for cohort studies [55].

### Study design

This is a retrospective, longitudinal, population-based cohort study that includes repeated measures at baseline and one year later.

### Setting

Healthcare in Israel is universal, and participation in a medical insurance plan is compulsory. All Israeli residents are entitled to basic health care as a fundamental right. The Israeli healthcare system is based on the National Health Insurance Law of 1995 [56], which mandates that all citizens residing in the country join one of four official health insurance organizations, all of which are run as not-for-profit organizations. The Meuhedet HMO is Israel’s third-largest integrated healthcare service provider, serving over 1.3 million patients nationwide of all ages. Patient-level data are stored by Meuhedet in a comprehensive data warehouse, including chronic illnesses, community-care visits, medications, laboratory test results, pharmaceutical records, and socio-demographic information. The frailty level of all HMO members aged 65+ is updated each month based on the electronic medical record. The data for this study were extracted from the Meuhedet Electronic Health Record on 1 January 2023 and 1 January 2024 to enable one-year follow-up.

### Eligibility criteria

The cohort included all 119,952 Meuhedet members aged 65 years and over who were alive at the beginning of 2023, including 2,811 who died during 2023, excluding those who left the HMO during 2023. Housebound individuals were included, but patients living in an institution were excluded since most of the medical information is filed in the institution and not in the HMO. Specifically, for the analysis of what will worsen first, the 2,811 patients who died before the end of the year were excluded as the presence of new deficits could not be assessed.

### Variables

#### Predictors

- Age groups: Age was divided into three categories: Young-old (65–74), middle-old (75–84), and oldest-old (85+). These categories are common and are based on biological aspects of age.
- Sex: Males and females, as recorded in the electronic health record.
- Sector: About 75% of Israelis are Jews, and one-quarter are Arabs, including Druze and Christian Arabs [57]. Among the Jewish population, about 17% are considered ultra-orthodox. Since the individual sector characteristic is not documented in the medical file, the sector used here is determined according to the clinic’s sector where the patient belongs, namely, the Jewish secular, the Jewish Orthodox, and the Arabic sectors. Since most of the clinics are located in neighborhoods mostly composed of members of only one sector, this method allows for adequate classification.
- Socio-economic status (SES): Derived from the individual’s home address and based on characteristics routinely collected by the Central Bureau of Statistics, ranging from 1 to 10. SES was grouped into three levels: 1–4 low, 5–7 medium, and 8–10 high.
- MEFI: MEFI, which stands for Meuhedet Electronic Frailty Index, is an EFI version we developed [6], based on Clegg [12] and on Orkaby Electronic Frailty Index [8]. MEFI is computed by extracting routinely collected health data directly from electronic medical records. It summarizes the number of deficits from a list of 36 variables, including chronic diseases, basic and instrumental activities of daily living, social aspects, mood, hearing or vision impairment, and cognitive functioning (see Table 1). The weight was the same for all the deficits, one point, conforming to Clegg’s definition. The look-back period for chronic diseases was from the age of 55, and the look-back period for non-disease deficits (such as functional deficits) was reduced to one year, a period that is well-accepted in the literature. Except for chronic diseases, a deficit that didn’t appear anymore in the electronic health record was considered to reflect recovery or resolution of the condition. This assumption is justified by the fact that the health system in Israel allows access to primary care at almost no cost, and indeed, only 2.3% did not visit any medical staff during the look-back period. The MEFI classifies individuals as ‘fit’ or exhibiting frailty in the ‘mild’, ‘moderate’, or ‘severe’ frailty range, based on the MEFI score (fit (0–0.12; 0–4 deficits), mild (0.13–0.24; 5–8 deficits), moderate (0.25–0.36; 8–12 deficits), and severe (>0.36; 13+ deficits), in line with EFI categories described in the literature [12, 14]. MEFI was shown to predict mortality and hospitalization [6]. More details of the validation study have been described elsewhere [6].
- CCI: The Charlson Comorbidity Index (CCI) assesses comorbidity levels by considering both the number and severity of 17 pre-defined comorbid conditions [58]. The higher the score, the higher the predicted mortality rate. CCI was categorized into four grades: no comorbidity (0), mild (1–2), moderate (3–5), and severe (6+). Five CCI comorbidities out of 19 were common to both CCI and MEFI.
- BMI: The BMI is based on the last height and weight measures recorded in the electronic health record in the HMO. It was categorized into four levels according to the division mostly used in health: underweight - less than 18.5, normal 18.5–<25, overweight 25–<30, and obese 30+.
- Hospitalization in the past year: This variable receives a value of 1 if the patient experienced any hospitalization in 2022, the year just preceding the follow-up period.

### Outcome measure

The frailty index is calculated every month. Worsening was defined as any change to a worse frailty category one year following diagnosis. The worsening outcome received a value of 1 if the MEFI level on 1 January 2024, as divided into four categories, was worse than the MEFI level on 1 January 2023. Those who passed away during 2023 received a value of 1, which is considered worsening.

For determining what worsened first, the new deficit measure received a value of 1 if the deficit didn’t appear in 2023 and appeared in 2024.

### Statistical methods

Descriptive statistics of the population were presented as either means (standard deviations) for continuous variables or percentages for categorical variables. Worsening was presented by percentages, overall, and stratified by MEFI. Comparing worsening by demographic and clinical characteristics was tested using the chi-square test. Additionally, multivariable logistic regression was conducted to identify variables associated with frailty worsening, and a concordance index (C-Index) was used for model validation. Multicollinearity was tested by calculating variance inflation factors (VIFs). The percentage of new deficits among those still alive at the end of the follow-up was presented as a percentage and sorted by decreasing size in each frailty group. Data were analyzed using IBM SPSS statistics software [59]. All statistical tests were two-sided, and *p*-values lower than 0.05 were considered statistically significant.

## Supplementary Materials

Supplementary Table 1
