# Supplementary material for: Frailty transitions in electronic health records: who first? what first?
Source: Aging (Albany NY). 2025 May 12;17(5):1148–63. doi: 10.18632/aging.206247 (PMC12151512; doi:10.18632/aging.206247)
Supplement: Supplementary Table 1 [file aging-17-206247-s001.pdf]

SUPPLEMENTARY MATERIALS

Supplementary Table

Supplementary Table 1. Top 15 deficits by MEFI and age groups.

		**FIT**	
All age groups	65–74	75–84	85+
30_Polypharmacy	30_Polypharmacy	30_Polypharmacy	18_Hearing
18_Hearing	18_Hearing	18_Hearing	16_Gait
16_Gait	26_MuscularWasting	16_Gait	30_Polypharmacy
26_MuscularWasting	16_Gait	26_MuscularWasting	20_Housebound
2_Anemia	2_Anemia	7_VisionComorbidity	26_MuscularWasting
7_VisionComorbidity	7_VisionComorbidity	2_Anemia	14_FallRelated
21_Hypertension	21_Hypertension	21_Hypertension	32_RequireForCare
33_Sleep disturbance	3_Anxiety	33_Sleep disturbance	1_ActLimitation
5_Arthritis	5_Arthritis	14_FallRelated	2_Anemia
3_Anxiety	33_Sleep disturbance	5_Arthritis	33_Sleep disturbance
15_Fatigue	15_Fatigue	22_Incontinence	7_VisionComorbidity
22_Incontinence	22_Incontinence	25_Memo_cog	34_SocialVulnerability
14_FallRelated	14_FallRelated	3_Anxiety	22_Incontinence
25_Memo_cog	36_WeightLoss	34_SocialVulnerability	15_Fatigue
36_WeightLoss	4_Depression	15_Fatigue	36_WeightLoss
		**MILD**	
All age groups	65–74	75–84	85+
30_Polypharmacy	30_Polypharmacy	30_Polypharmacy	30_Polypharmacy
18_Hearing	18_Hearing	18_Hearing	20_Housebound
16_Gait	16_Gait	16_Gait	18_Hearing
26_MuscularWasting	26_MuscularWasting	26_MuscularWasting	16_Gait
2_Anemia	2_Anemia	2_Anemia	26_MuscularWasting
33_Sleep disturbance	33_Sleep disturbance	33_Sleep disturbance	14_FallRelated
14_FallRelated	22_Incontinence	14_FallRelated	2_Anemia
22_Incontinence	3_Anxiety	22_Incontinence	1_ActLimitation
20_Housebound	14_FallRelated	20_Housebound	22_Incontinence
7_VisionComorbidity	7_VisionComorbidity	7_VisionComorbidity	33_Sleep disturbance
3_Anxiety	5_Arthritis	25_Memo_cog	25_Memo_cog
25_Memo_cog	15_Fatigue	3_Anxiety	7_VisionComorbidity
5_Arthritis	20_Housebound	15_Fatigue	34_SocialVulnerability
15_Fatigue	25_Memo_cog	1_ActLimitation	32_RequireForCare
4_Depression	21_Hypertension	34_SocialVulnerability	36_WeightLoss
		**MODERATE**	
All age groups	65-74	75–84	85+
16_Gait	30_Polypharmacy	16_Gait	20_Housebound
30_Polypharmacy	16_Gait	26_MuscularWasting	16_Gait
26_MuscularWasting	26_MuscularWasting	30_Polypharmacy	26_MuscularWasting
18_Hearing	18_Hearing	18_Hearing	30_Polypharmacy

20_Housebound	2_Anemia	20_Housebound	18_Hearing
2_Anemia	22_Incontinence	2_Anemia	14_FallRelated
14_FallRelated	33_Sleep disturbance	14_FallRelated	2_Anemia
22_Incontinence	20_Housebound	22_Incontinence	1_ActLimitation
33_Sleep disturbance	14_FallRelated	33_Sleep disturbance	22_Incontinence
1_ActLimitation	4_Depression	1_ActLimitation	33_Sleepdisturbance
7_VisionComorbidity	1_ActLimitation	4_Depression	25_Memo_cog
4_Depression	7_VisionComorbidity	7_VisionComorbidity	7_VisionComorbidity
25_Memo_cog	3_Anxiety	25_Memo_cog	34_SocialVulnerability
3_Anxiety	15_Fatigue	3_Anxiety	4_Depression
36_WeightLoss	25_Memo_cog	36_WeightLoss	36_WeightLoss
**SEVERE**			
All age groups	65–74	75–84	85+
16_Gait	16_Gait	16_Gait	20_Housebound
20_Housebound	26_MuscularWasting	26_MuscularWasting	16_Gait
26_MuscularWasting	20_Housebound	20_Housebound	26_MuscularWasting
14_FallRelated	2_Anemia	2_Anemia	14_FallRelated
2_Anemia	14_FallRelated	14_FallRelated	2_Anemia
18_Hearing	21_Hypertension	22_Incontinence	18_Hearing
22_Incontinence	33_Sleep disturbance	18_Hearing	1_ActLimitation
1_ActLimitation	22_Incontinence	1_ActLimitation	33_Sleep disturbance
33_Sleep disturbance	18_Hearing	33_Sleep disturbance	22_Incontinence
4_Depression	1_ActLimitation	4_Depression	21_Hypertension
21_Hypertension	4_Depression	36_WeightLoss	34_SocialVulnerability
25_Memo_cog	3_Anxiety	3_Anxiety	7_VisionComorbidity
36_WeightLoss	25_Memo_cog	25_Memo_cog	25_Memo_cog
7_VisionComorbidity	7_VisionComorbidity	34_SocialVulnerability	36_WeightLoss
34_SocialVulnerability	15_Fatigue	7_VisionComorbidity	4_Depression
